# Inhibition of serine/threonine protein phosphatase PP1 protects cardiomyocytes from tunicamycin-induced apoptosis and I/R through the upregulation of p-eIF2α

**DOI:** 10.3892/ijmm.2013.1603

**Published:** 2013-12-23

**Authors:** CHUN-LEI LIU, YUN-YUN HE, XIN LI, RUI-JUN LI, KUN-LUN HE, LI-LI WANG

**Affiliations:** 1 Department of Cardiology, Chinese PLA General Hospital, Beijing 100853, P.R. China; 2 Medical School of Nankai University, Tianjin 300071, P.R. China; 3 Beijing Institute of Pharmacology and Toxicology, Beijing 100850, P.R. China

**Keywords:** serine/threonine protein phosphatase PP1, PP1-12 inhibitor, ER stress, ischemia/reperfusion injury, cardiovascular disease, apoptosis

## Abstract

The serine/threonine protein phosphatase PP1 mediates the dephosphorylation of phosphorylated eukaryotic translation initiation factor 2 subunit α (p-eIF2α), which is a central regulator of protein synthesis. In the present study, we examined the protective effects of PP1–12 (an inhibitor of the serine/threonine protein phosphatase PP1) against tunicamycin (TM)-induced apoptosis in cultured cardiomyocytes *in vitro*, as well as in an *in vivo* model of ischemia/reperfusion (I/R) injury in rat hearts. Neonatal cardiomyocytes cultured from the ventricles of the hearts of 1-day-old Wistar rats were exposed to various concentrations of PP1–12 (0.3, 1 and 3 μmol/l) for 30 min, followed by treatment with TM for 36 h. Cell viability was assessed by adenosine triphosphate (ATP) bioluminescence, and the results revealed that pre-treatment with PP1–12 protected cell viability. Western blot analysis revealed that PP1–12 induced eIF2α phosphorylation and immuncytochemistry indicated that PP1–12 downregulated the expression of C/EBP homologous protein (CHOP), which is related to apoptosis. PP1–12 suppressed cell apoptosis, with maximum protective effects displayed at the concentration of 3 μmol/l. For the *in vivo* experiments, male Sprague-Dawley rats were randomly divided into 5 groups: i) sham-operated; ii) vehicle (I/R + DMSO); iii) I/R + 1 mg/kg/day PP1–12; iv) I/R + 3 mg/kg/day PP1–12; and v) I/R + 10 mg/kg/day PP1–12. PP1–12 reduced the expression of cleaved caspase-12 and increased the phosphorylation of eIF2α, as revealed by western blot analysis. By calculating the apoptotic index (AI), we found that 10 mg/kg/day PP1–12 exerted the most pronounced anti-apoptotic effect. The infarction area was significantly decreased following treatment with this concentration of PP1–12, as revealed by 2,3,5-triphenyltetrazolium chloride (TTC) staining. Taken together, these data suggest that PP1–12 protects cardiomyocytes from TM- and I/R-induced apoptosis, and this effect is achieved at least in part through the inhibition of cell apoptosis and the induction of eIF2α phosphorylation.

## Introduction

Protein phosphorylation and dephosphorylation at serine (Ser)/threonine (Thr) residues is a central biochemical reaction that is regulated by Ser/Thr kinase and phosphatase activities. While much attention has been paid to the roles of different kinases, little is known about the role of phosphatases (1). The Ser/Thr protein phosphatase PP1 and its non-enzymatic co-factor, growth arrest and DNA damage-inducible protein 34 (GADD34), can mediate the dephosphorylation of phosphorylated eukaryotic translation initiation factor 2 subunit α (p-eIF2α), which is a key factor in the regulation of protein synthesis (2,3). Therefore, the Ser/Thr protein phosphatase PP1 is considered an important cellular target of protein synthesis.

In 2005, Boyce *et al* reported that a small molecule, named salubrinal, selectively inhibited the PP1/GADD34-mediated dephosphorylation of p-eIF2α, thereby protecting 36% of PC12 cells from endoplasmic reticulum (ER) stress-induced apoptosis, with cell viability increasing from approximately 40 to 76% (4). In a previous study by our group, we confirmed the ability of salubrinal to protect cardiomyocytes from apoptosis (5). However, salubrinal is still far from being the ideal cardioprotective therapeutic agent due to its side-effects, poor solubility and high effective concentration. Apart from salubrinal, very few substances that can potentially protect cardiomyocytes are known.

A structure-activity relationship (SAR) study on salubrinal was previously carried out by our group (6) in search of potent cardioprotective agents derived from this molecule. We demonstrated that the trichloromethyl and cinnamide groups of salubrinal are indispensable for its activity, whereas the quinoline ring terminus is a key site for modification. We therefore modified the quinoline ring terminus, as well as the thiourea group, and synthesized a total of 95 molecules. Through screenings with cell viability assays, we focused on the small molecule, PP1–12. In the present study, we evaluated the ability of this molecule to protect neonatal cardiomyocytes *in vitro* from apoptosis induced by tunicamycin (TM) and ischemia/reperfusion (I/R) injury. In addition, we aimed to determine its effects on myocardial I/R injury in rat hearts *in vivo*.

## Materials and methods

### Cell culture and treatments

Neonatal cardiomyocytes were isolated from the ventricles of the hearts of 1-day-old Wistar rats (Academy of Military Medical Sciences, Beijing, China, License no. SCXK-2007-004). Briefly, the rats were sacrificed and the hearts excised. Following homogenization with a scalpel, the hearts were treated with 0.125% trypsin and 0.1% collagenase II (4:1) at 37°C for 10 min; 4–5 rounds of digestion were performed. Isolated cells were collected by centrifugation and resuspended in Dulbecco’s modified Eagle’s medium (DMEM) containing 10% fetal bovine serum (FBS), 100 U/ml penicillin and 100 μg/ml streptomycin. The cultures were enriched with myocytes by pre-plating for 90 min to deplete the non-myocyte population. Non-attached cells were plated onto plastic culture dishes at the appropriate cell density. The cells were cultured at 37°C in 95% air-5% CO_2_ for 24 h, and then half of the DMEM medium containing 10% FBS was replaced with 0.1% 5-bromo-2-deoxyuridine (BrdU). The culture medium was changed every 48 h. After 4 days, the culture medium was changed to fresh DMEM containing 1% FBS and the cells were cultured for another 24 h. Subsequently, the cells were treated with TM. The medium was changed to fresh DMEM containing 1% FBS 1 h prior to exposure to reagents, in order to obtain consistent ER stress response data. All reagents were obtained from Gibco-BRL (Gaithersburg, MD, USA), unless otherwise stated.

### Cell viability assay

Adenosine triphosphate (ATP) bioluminescence was used as a marker of cell proliferation and viability. The presence of ATP was estimated using the CellTiter-Glo^®^ Luminescent Cell Viability Assay kit (Promega Corp., Madison, WI, USA) following the manufacturer’s instructions. Briefly, purified neonatal rat cardiomyocytes were inoculated into 96-well plates (10^4^/well) and treated with various concentrations of TM dissolved in DMSO (Sigma, St. Louis, MO, USA). Various concentrations of PP1–12 (0.01, 0.03, 0.1, 0.3, 1, 3, 10 μmol/l) were added 30 min prior to treatment with TM. Subsequently, a volume of CellTiter-Glo reagent equal to the volume of the cell culture medium present in each well was added, and the contents were mixed for 2 min in an orbital shaker to induce cell lysis. The wells were incubated at room temperature for 10 min to stabilize the luminescent signal.

### Immunocytochemistry

Purified neonatal rat cardiomyocytes (10^4^/well) were inoculated into 96-well assay plates (black plates with clear bottom). Various concentrations of PP1–12 (0.1, 0.3, 1 μmol/l) were added 30 min prior to treatment with TM. Cells were fixed in 4% paraformaldehyde for 15 min at room temperature, rinsed with PBS, and incubated with blocking buffer (1X PBS, 0.3% Triton X-100) supplemented with 5% serum from the same species as the secondary antibody, for 1 h at room temperature. Then, blocking solution was removed, primary mouse monoclonal antibody against C/EBP homologous protein (CHOP)/growth arrest and DNA damage-inducible protein 153 (GADD153) from Cell Signaling Technology (1:1,600 dilution) was added, and the cells were incubated overnight at 4°C. Subsequently, 3 μM goat anti-mouse IgG (H&L) secondary antibody conjugated with fluorochromes Hoechst 33258 (nuclear dye) or DyLight™ 594 (1:500; Thermo Fisher Scientific, Waltham, MA, USA) were added, followed by incubation for 1 h at room temperature in the dark. The secondary antibody was aspirated and the wells were washed 3 times with PBS. The cells were then scanned on an In Cell Analyzer 2000 high-content screening (HCS) reader (General Electric, Atlanta, GA, USA).

### Multiparametric analysis of apoptosis

Cellomics^®^ Multiparameter Apoptosis kits were purchased from Thermo Fisher Scientific. Multiparametric analysis of apoptosis was carried out using Hoechst dye, Alexa Fluor 488 conjugated to phalloidin and MitoTracker, as previously described (7). Purified neonatal rat cardiomyocytes (10^4^/well) were inoculated into 96-well assay plates (black plates with clear bottom). Various concentrations of PP1–12 (0.3, 1, 3 μmol/l) were added 30 min prior to treatment with TM. At 30 min before the end of the incubation period, we added MitoTracker/Hoechst solution followed by incubation for an additional 30 min at 37°C. Fixation solution was added without removing the medium and the cells were incubated under a fume hood at room temperature for 10 min. After rinsing with PBS, the cells were permeabilized with permeabilization buffer and incubated for 15 min. The cells were then incubated with Alexa Fluor 488 phalloidin solution for 30 min at room temperature in the dark. The solution was aspirated and the cells were washed 3 times with PBS. The data were evaluated on an In Cell Analyzer 2000 HCS reader.

### Rat models of I/R injury

Adult male Sprague-Dawley rats weighing 200–250 g were obtained from the Charles River Company of China (License no. SCXK-2011-0004). All experiments were performed in accordance with the ethical regulations of the Institutional Animal Care and Use of Laboratory Animals of the Chinese PLA General Hospital. Fifty rats were randomly divided into 5 experimental groups: i) the sham-operated group; ii) the vehicle-treated group (I/R + DMSO); iii) the I/R + S1 group (I/R +1 mg/kg/day PP1–12); iv) the I/R + S3 group (I/R +3 mg/kg/day PP1–12); and v) the I/R + S10 group (I/R + 10 mg/kg/day PP1–12). PP1–12 was dissolved in DMSO and administered by intraperitoneal injection once a day for 3 consecutive days. The final injection was administered 30 min prior to the coronary occlusion. The rat model of I/R injury was established by ligation of the left anterior descending coronary artery (LAD). The heart was exposed via a left thoracotomy and the left coronary artery was ligated 2–3 mm from its original location between the conus of the pulmonary artery and the left atrium, using a 6-0 silk suture. An inflatable balloon of fixed atmosphere was placed between the artery and the silk suture. The coronary artery was occluded to induce ischemia for 30 min, followed by deflation of the balloon to allow coronary reperfusion for 2 h. In the sham-operated group, the suture around the coronary artery was not tied.

### Western blot analysis

*In vitro*, purified neonatal cardiomyocytes (10^6^/well) were inoculated into 6-well plates and treated with various concentrations of PP1–12 (0.1, 0.3, 1 μmol/l) 30 min prior to treatment with TM. *In vivo*, the rats were sacrificed and the hearts excised and cut into into 50-mg sections and frozen in liquid nitrogen. Primary cells and tissue scraps were lysed in whole-cell lysis buffer [62.5 mM Tris-HCl (pH 6.8 at 25°C), 2% w/v SDS, 10% glycerol, 50 mM DTT]. The homogenates were heated at 100°C for 10 min, and centrifuged at 12,000 × g for 10 min at 4°C. Tissue extracts (50 μg of protein) were homogenized in lysis buffer (50 mM Tris-HCL, pH 7.5, 150 mM NaCl, 1% Nonidet P-40, 0.5% sodium deoxycholate) with protease inhibitors and a phosphatase inhibitor. Following centrifugation at 13,000 × g, the supernatants were boiled for 5 min in Laemmli loading buffer. The supernatants were used as protein samples. We used BCA to determine the protein concentration of each sample. Cellular proteins were separated by electrophoresis on 10% SDS polyacrylamide gels and transferred onto PVDF membranes. After blocking (in 1X TBS, 0.1% Tween-20 with 5% w/v non-fat dry milk), the membranes were incubated overnight in gentle agitation mode at 4°C with the following antibodies: rat monoclonal anti-caspase-12 and anti-GAPDH (1:200 dilution; Santa Cruz Biotechnology, Inc., Santa Cruz, CA, USA), and rabbit polyclonal anti-phospho-eIF2α, anti-eIF-2α and anti-β-actin (1:200 dilution; Cell Signaling Technology). This was followed by incubation with the appropriate horseradish peroxidase-conjugated secondary antibodies (1:5,000 dilution; Beijing Zhongshan Golden Bridge Biotechnology Co., Beijing, China) for 1 h at room temperature. The film was scanned and analyzed with an imaging densitometer (AlphaImager FluorChem 5500, Alpha Innotech Corp., San Leandro, CA, USA).

### Terminal deoxynucleotidyl transferase dUTP nick end-labeling (TUNEL) assay

Hearts fixed in 10% formalin were embedded in paraffin and sectioned into 4-μm-thick slices. To detect apoptotic cells, TUNEL labeling was performed using an In Situ Apoptosis Detection kit (Roche Diagnostics GmbH, Basel, Switzerland). To analyze the number of apoptotic cells in the infarcted hearts, digital images were acqired at ×400 magnification, and 5 random fields from each sample were quantified. We counted the number of TUNEL-positive cardiomyocytes and hematoxylin-stained nuclei in the entire section of each sample.

### 2,3,5-Triphenyltetrazolium chloride (TTC) staining

The hearts were rapidly excised, placed in a 0.9% saline solution, and cut transversally from the apex to the base into 5 slices of equal thickness (2.0 mm). The slices were incubated for 10 min in phosphate-buffered 1% TTC at 37°C, then fixed in 10% formalin solution. The area that was not stained by TTC was the area of infarction, and was assessed by a blinded observer using computer-assisted planimetry (NIH Image 1.57 software, available at: http://rsb.info.nih.gov/nih-image). The percentage of infarction was expressed as the ratio of the infarct size (IS, or area of infraction) to the ventricle size (VS).

### Statistical analysis

Each experiment was performed in a minimum of 3 different cultures and was repeated at least 3 times. The presented data are a compilation of these repetitions, apart from the western blot images, which are from one representative of the 3 replicate experiments. Values are shown as the means ± standard deviation (SD). Statistical analyses were performed by a one-way analysis of variance (ANOVA). Values of P<0.05 were considered to indicate statistically significant differences.

## Results

**Figure 1:**
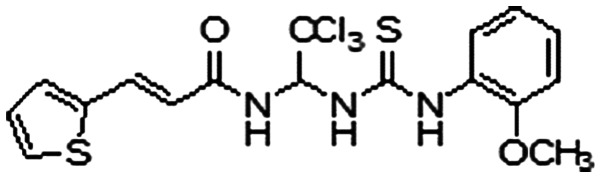
Chemical structure of PP1–12: (E)-3-(Thiophen-2-yl)-N-(2,2,2-trichloro-1-(3-(2-methoxyphenyl)thioureido)ethyl)acrylamide.

**Figure 2:**
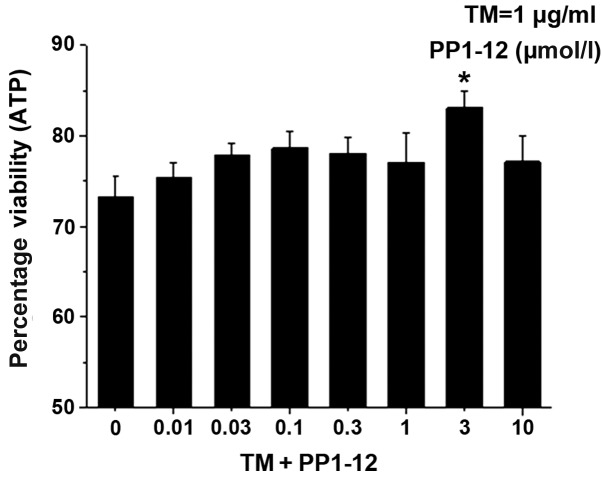
Protective effect of various concentrations of PP1–12 on cardiomyocytes treated with tunicamycin (TM) for 36 h, assessed by cellular adenosine triphosphate (ATP) content. Error bars represent the means ± SD. ^*^p<0.05 for the comparison between TM + PP1–12 (3 μmol/l) and the TM group treated with TM only.

In a previous study, through the screening of 95 compounds designed as inhibitors of Ser/Thr protein phosphatase PP1, we identified a small molecule with a low half maximal effective concentration (EC_50_) and almost zero toxicity (6). The chemical structure of this compound, named PP1–12, is shown in Fig. 1. PP1–12 protected the viability of the cells treated with the protein glycosylation inhibitor, TM, in a dose-dependent manner, with 3 μmol/l assessed as the most effective concentration, through the calculations of the ATP content (Fig. 2).

**Figure 3:**
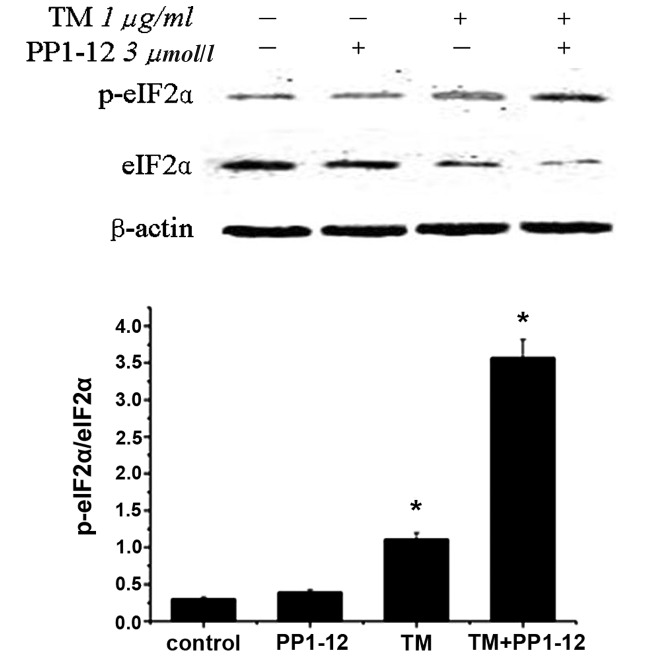
PP1–12 increases the phosphorylation of eukaryotic translation initiation factor 2 subunit α (eIF2α) in cardiomyocytes after 4 h of treatment with tunicamycin (TM). The protein level of p-eIF2α was analyzed by western blot analysis. Control, treated with DMSO; TM, vehicle-treated group (TM + DMSO). ^*^P<0.05 for the comparison between the TM-treated groups and the control.

**Figure 4:**
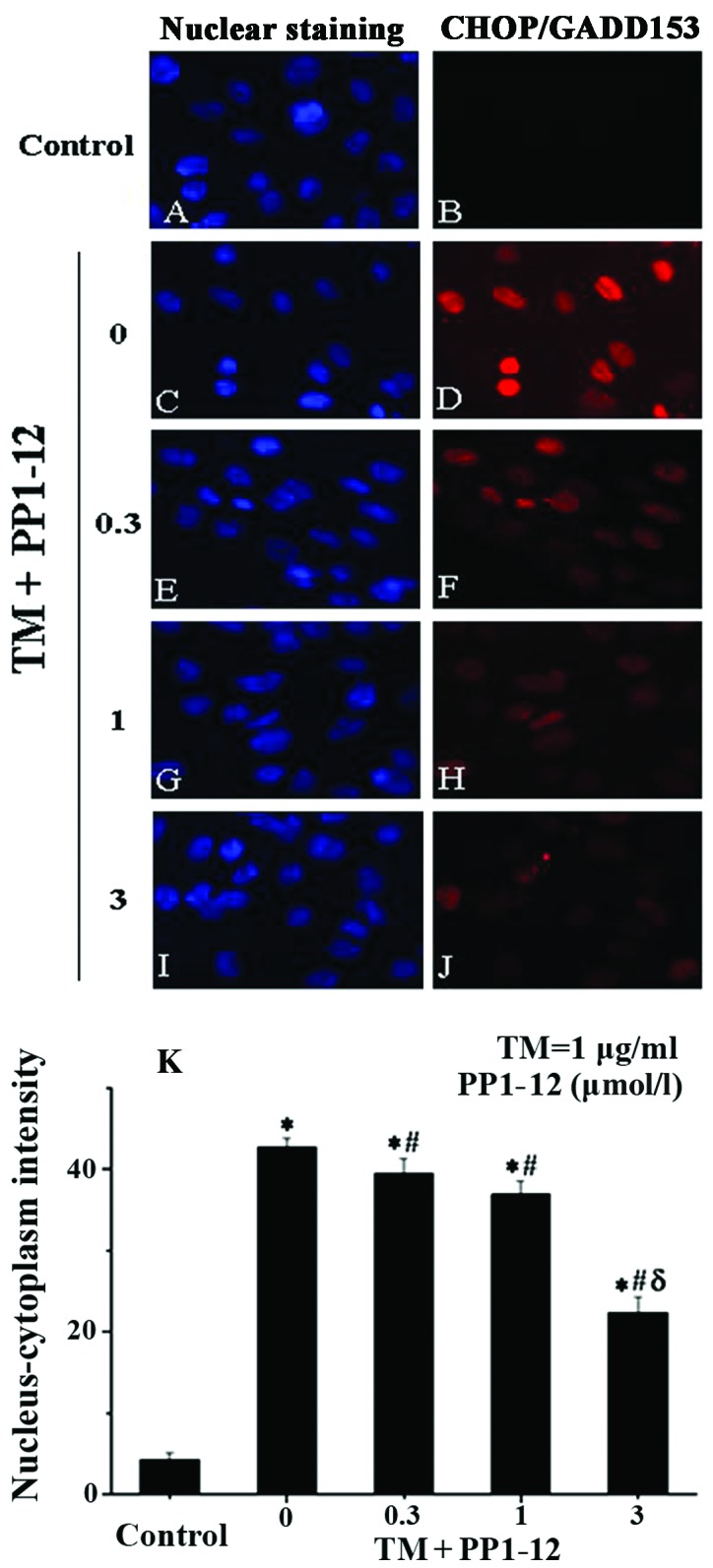
PP1–12 treatment reduces the tunicamycin (TM)-induced expression of CHOP in cardiomyocytes, as assessed by high-content analysis (HCA). (A, C, E, G and I) Nuclear staining with Hoechst. (B, D, F, H and J) Staining of CHOP/GADD153. (K) Relative CHOP presence in the nucleus/cytoplasm. ^*^P<0.001 for the comparison between TM-treated groups and the control group (DMSO); ^#^P<0.05 for the comparison between TM groups treated with various PP1–12 concentrations and the TM group treated with DMSO (0. μmol/l PP1–12).

TM inhibits protein glycosylation in the ER, thereby inducing ER stress. Apoptosis is induced by severe and prolonged ER stress (8). PP1–12 was designed as an inhibitor of Ser/Thr protein phosphatase PP1, targeting the phosphatase activity of eIF2α and thereby, reducing ER-related apoptosis. In this study, the level of eIF2α phosphorylation was detected by western blot analysis. Notably, p-eIF-2α/eIF-2α was slightly increased in the TM-treated cells. As expected, the p-eIF-2α/eIF-2α level considerably increased with PP1–12 pre-treatment (Fig. 3). PP1–12, as a protein phosphatase inhibitor, is known to induce the robust phosphorylation of eIF2α (2,6). The CHOP protein is specific to ER stress-induced apoptosis, with its expression induced by severe and prolonged ER stress. To examine the association betweem PP1–12, ER stress and apoptosis, we examined CHOP expression by immunocytochemistry (9), as previously described. We observed that CHOP was present at relatively high levels in the TM-treated cells, while its relative expression (nucleus/cytoplasm-detected intensity) was reduced with PP1–12 pre-treatment in a dose-dependent manner (Fig. 4), decreasing from 40% in the TM-treated cells to 20% in the cells treated with TM + 3 μmol/l PP1–12.

**Figure 5:**
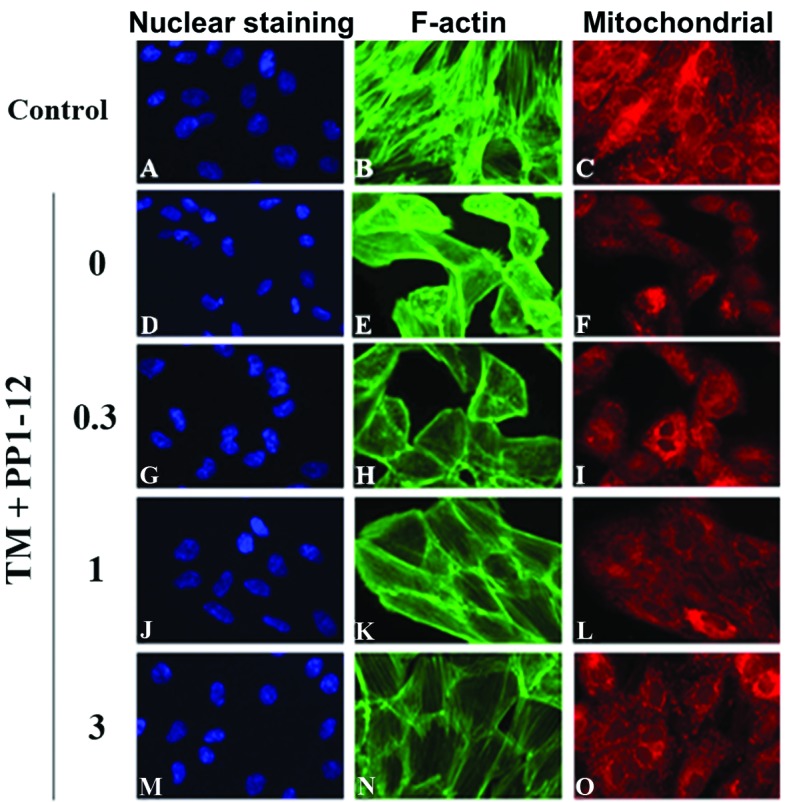

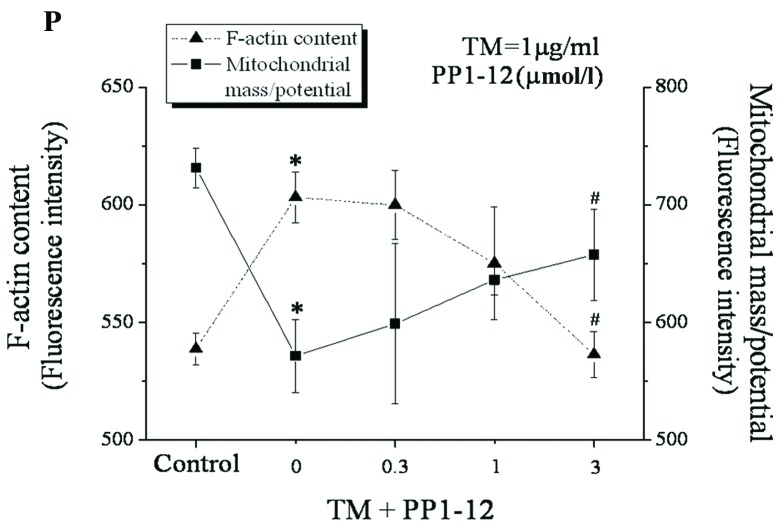
PP1–12 reduces apoptosis in cardiomyocytes treated with tunicamycin (TM) for 36 h, as assessed by high-content analysis (HCA). (A, D, G, J and M) Nuclear staining with Hoechst. (B, E, H, K and N) Staining for F-actin. (C, F, I, L and O) Mitochondrial staining. (P) F-actin content and mitochondrial mass/potential ratio. ^*^P<0.001 for the comparison between the TM group treated with DMSO (0. μM PP1–12) and the control group (DMSO); ^#^P<0.001 for the comparison between the TM group treated with 3 μmol/l PP1–12 and the TM + DMSO group.

We then examined the anti-apoptotic effects of PP1–12 by the multiparametric analysis of apoptosis. The cells displayed significant nuclear condensation, a decrease in mitochondrial membrane potential and an increase in actin cytoskeleton content following exposure to TM (1 μg/ml) for 36 h, as compared to the control group, treated with DMSO. Apoptosis was inhibited by PP1–12 (0.3, 1 and 3 μmol/l), as shown by a reduction in nuclear condensation (Fig. 5A, D, G, J and M), but also, by the increase in mitochondrial membrane potential (Fig. 5B, E, H, K and N) and the decrease in actin cytoskeleton content (Fig. 5C, F, I, L and O); these changes were significant at a concentration of 3 μmol/l PP1–12 (Fig. 5P).

**Figure 6:**
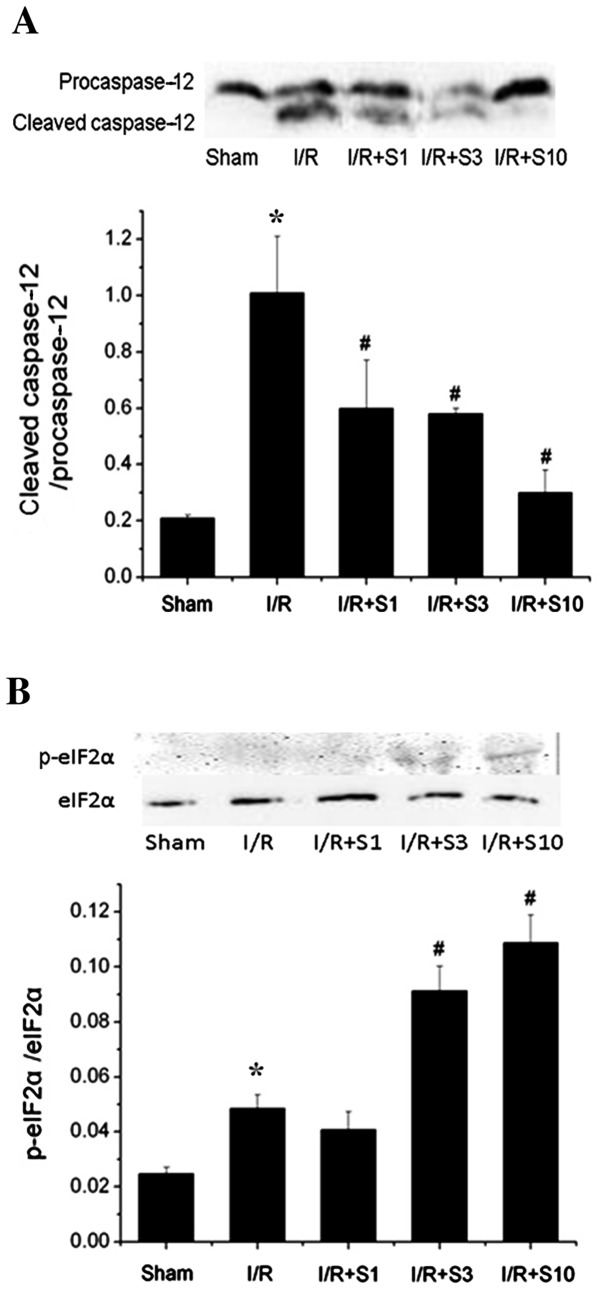
Immunoblot analysis and relative ratios of (A) cleaved caspase-12/caspase-12 (procaspase-12) and (B) p-eIF2α/eIF2α in a rats with ischemia/reperfusion (I/R) injury model treated with 1 (I/R + S1), 3 (I/R + S3) and 10 mg/kg/day (I/R + S10) of PP1–12. ^*^P<0.05 vs. the sham-operated group; ^#^P<0.05 vs. the I/R (vehicle-treated group, DMSO) group (n=6).

To examine whether the addition of PP1–12 has a protective effect on the heart under pathological stress, we selected a widely used model of I/R. We induced ischemia for 30 min and then reperfusion injury for 2 h. The expression of p-eIF2α and cleaved caspase-12 (the ER stress-specific apoptotic protein) was detected by western blot analysis. Caspase-12 was activated by I/R, as demonstrated by an increase in the level of the cleaved caspase-12/caspase-12 ratio. The activation of caspase-12 was largely reverted by PP1–12. A decrease by up to 63.47% was observed upon PP1–12 pre-treatment (10 mg/kg/day) compared to the I/R (vehicle-treated) group (Fig. 6A). Similar to the *in vitro* assays, the level of p-eIF2α was slightly increased in the I/R (vehicle-treated, I/R + DMSO) group compared to the control (sham-operated) group, and there was a significant increase in p-eIF2α expression (Fig. 6B) in the rats pre-treated with PP1–12 (10 mg/kg/day).

**Figure 7:**
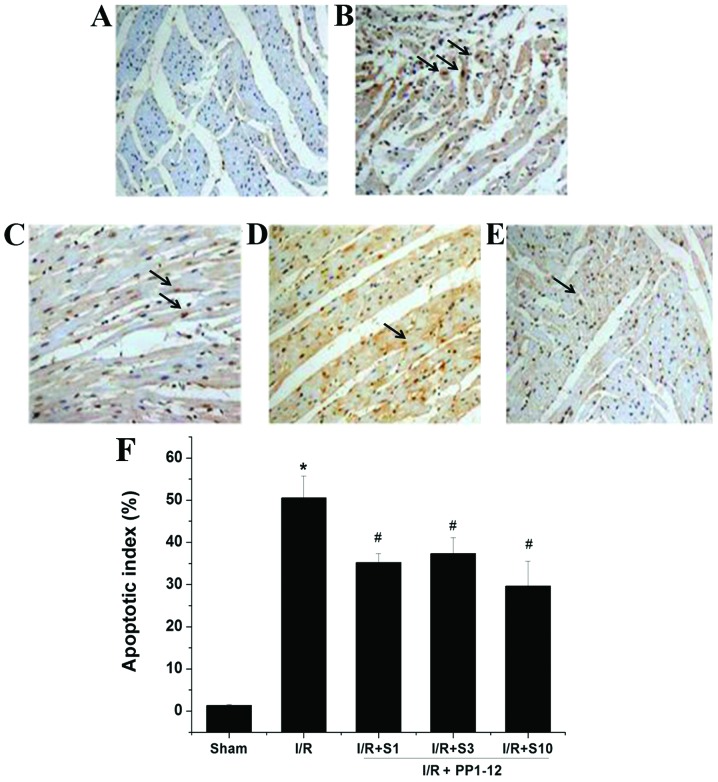
Treatment with PP1–12 attenuates ischemia/reperfusion (I/R) injury-induced myocardial apoptosis. TUNEL assay (x400 magnification) on (A) sham-operated, (B) I/R (vehicle-treated,. DMSO), (C) I/R + S1 (1 mg/kg/day), (D) I/R + S3 (3 mg/kg/day) and (E) I/R + S10 (10 mg/kg/day) groups. (F) Mean percentage of apoptotic cells (apoptotic index) for the same groups. I/R + S1, S3 and S10, I/R treated with 1, 3 and 10 mg/kg/day PP1–12, respectively. Results are shown as the means ± SD. ^*^P<0.05 vs. the sham-operated group; ^#^P<0.05 vs. the I/R (vehicle-treated, DMSO) group. Black arrows indicate the TUNEL-positive cells.

TUNEL is a method widely used for the detection of apoptosis, whereby apoptotic cells are distinguished by their color. The number of TUNEL-positive cells was significantly increased in the hearts of rats in the I/R (vehicle-treated) group compared with the sham-operated group (Fig. 7B). Treatment with PP1–12 markedly and significantly reduced the number of TUNEL-positive cells. Among the doses of PP1–12 tested, that of 10 mg/kg/day had the most pronounced effect. At this dose, the number of TUNEL-positive cells was reduced by almost 10% compared to treatment with the vehicle (I/R group). No TUNEL-positive cells were observed in the sham-operated group (Fig. 7A).

**Figure 8:**
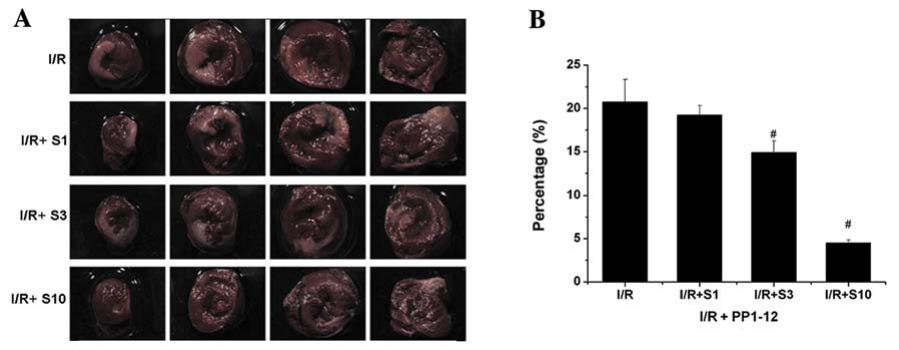
Effect of PP1–12 on ischemia/reperfusion (I/R) injury in rat hearts. (A) 1% 2,3,5-Triphenyltetrazolium chloride (TTC) staining of heart sections. (B) Percentage of infarction (infarct size/ventricle size). I/R + S1, I/R + S3 and I/R + S10 stand for 1, 3 and 10 mg/kg/day of PP1–12, respectively (n=6 in each group). ^#^P<0.05 vs. the I/R (vehicle-treated, DMSO) group.

The area of infarction is a vital index of myocardial cardiac injury. I/R can induce heart infarction by inducing cell apoptosis. The infarct size was measured using TTC cell staining and was expressed as the percentage ratio of the infarct size to the ventricle size (IS/VS). In the vehicle-treated group (I/R + DMSO), the percentage of IS/VS was approximately 20.738%. Following the administration of PP1–12, the I/R + S1, I/R + S3 and I/R + S10 groups (treated with 1, 3 and 10 mg/kg/day PPI-12, respectively) showed a decrease in the myocardial infarct size of 19.231, 14.917 (P<0.05) and 4.518% (P<0.05), respectively, as compared to the vehicle-treated group (Fig. 8).

## Discussion

Our study provides evidence on the anti-apoptotic effects of the inhibitor of the Ser/Thr protein phosphatase PP1, PP1–12, on cardiomyocytes, through the upregulation of p-eIF2α.

PP1, the major isotype of Ser/Thr protein phosphatases in cardiomyocytes, is a critical negative regulator of Ca^2+^ cycling and contractility. PP1 is a holoenzyme comprising catalytic and regulatory subunits (10). The inhibition of PP1 has been shown to enhance cardiac function and delay the progression of heart failure (11–13). Notably, previous studies have focused on the inhibition of the catalytic activity of PP1. In 2005, Boyce *et al* (4) found that salubrinal, a selective inhibitor of the regulatory subunit of PP1, protects PC-12 cells from apoptosis. They further demonstrated that salubrinal inhibits targeting, by PP1, of the eIF2α protein, which normally results in its dephosphorylation and allows proteostasis. In 2012, we demonstrated for the first time that salubrinal has a similar protective effect on cardiomyocytes (5). There are a number of studies focusing on protein phosphorylation, and all highlight the important effects of protein phosphorylation on protein expression. Protein phosphorylation can be regulated by the balance between kinase and phosphatase activities. While more attention has been paid to the roles of different kinases, less is known about the role of phosphatases (14). We previously synthesized a number of inhibitors of Ser/Thr protein phosphatase PP1 based on salubrinal, of which 24 displayed protective effects on cardiomyocytes (6). Viability assays revealed that, of these molecules, PP1–12 protects cardiomyocytes from TM-induced death. We thus focused on PP1–12, since it further displayed reduced EC_50_ and low toxicy.

Cardiomyocytes are cells with no proliferative ability. Therefore, the maintenance of their population through the prevention of apoptosis is crucial. Accumulating evidence suggests that ER stress-mediated cell apoptosis is one of the leading causes of cardiomyocyte death. TM is one of the most widely used compounds for triggering ER stress through the inhibition of protein glycosylation (14). ER stress in abnormal cardiomyocytes induces the so-called unfolded protein response (15), which involves p-eIF2α-mediated inhibition of protein translation, thereby protecting cells from ER sress. The dephosphorylation of p-eIF2α is regulated by a complex containing the Ser/Thr phosphatase PP1 and its co-factor, GADD34 (16). The apoptotic pathway is induced by severe and prolonged ER stress (17,8). PP1–12 was designed as an inhibitor of the PP1/GADD34 complex to mediate the dephosphorylation of p-eIF2α. As expected, western blot analysis revealed an increase in the p-eIF-2α/eIF-2α ratio upon PP1–12 pre-treatment. Of note, TM alone also induced a slight increase in this ratio, which possibly reflects a self-protective mechanism of cardiomyocytes against ER stress. CHOP is a key transcription factor acting downstream of p-eIF2α, whose expression is closely associated with the induction of ER-induced apoptosis (18). Our immunohistochemical analyses suggested that TM induces, while PP1–12 inhibits, CHOP transcription. This result further indicated that PP1–12 may reduce ER stress-related apoptosis. High-content screening (HCS), which allows fluorescence-based multiparametric analyses, has rapidly become a widely used method for the detection of apoptosis. The most obvious advantage of using this method is that it allows an image-based analysis. Moreover, HCS combined with a microplate reader, allows the analysis of a larger number of samples, entails the reduced consumption of reagents, and lower heterogeneity. In our study, HCS analysis revealed that PP1–12 protects cardiomyocytes from apoptosis, as shown by the reduction in nuclear condensation, the increase in mitochondrial membrane potential and the decrease in actin cytoskeleton content. Taken together, our data suggest that the protective effects of PP1–12 are at least partly mediated by a resistance to apoptosis. This result is consistent with the results of our previous study on the effects of salubrinal on cardiomyocytes (6).

In 2013, Oh *et al* demonstrated that decoy peptides target protein phosphatase 1 and inhibit the dephosphorylation of phospholamban in cardiomyocytes (19). In addition, Hanana *et al* pointed out that the activation of ERK by a potent inhibitor of protein phosphatase exerts a cell survival effect (20). Together with our results, these data demonstrate that PP1 may be a protective agent in cardiomyocyte injury.

A number of studies have demonstrated that ER stress response is activated in heart tissue exposed to prolonged and acute stress. I/R is a type of acute stress that can activate ER stress (21). In our model of I/R, p-eIF2α, as well as cleaved caspase-12 were activated. Caspase-12 is an apoptosis-specific protein, which can be cleaved only in ER stress-related apoptosis (22). In our study, PP1–12 significantly increased the expression of p-eIF2α and reduced the level of cleaved caspase-12. This result indicates that PP1–12 inhibits ER stress-related apoptosis. Thus, we concluded that PP1–12 may have an anti-apoptotic effect. Our hypothesis was further supported by the decrease in the number of TUNEL-positive cells pre-treated with PP1–12. The area of infarction is a vital index of heart injury in models of I/R (23). In our study, the infarct size decreased with the increasing concentrations of PP1–12. This indicates that PP1–12 exerts a protective effect in the rat model of I/R. Although the anti-apoptotic effects of PP1–12 may explain the protection of the cells in heart tissue, the underlying mechanisms which render PP1–12 a more potent protective agent compared to other inhibitors of Ser/Thr protein phosphatase PP1 remain unknown.

In conclusion, the data presented in this study demonstrate that PP1–12 protects cardiomyocytes from apoptosis. Thus, PP1–12, as an inhibitor of Ser/Thr protein phosphatase PP1, may thus represent a promising therapeutic agent for the control of heart disease. In the future, drugs that target Ser/Thr protein phosphatase PP1 may therefore complement the currently used clinical treatments.
